# Combined Antiviral Therapy Using Designed Molecular Scaffolds Targeting Two Distinct Viral Functions, HIV-1 Genome Integration and Capsid Assembly

**DOI:** 10.1038/mtna.2015.22

**Published:** 2015-08-25

**Authors:** Wannisa Khamaikawin, Somphot Saoin, Sawitree Nangola, Koollawat Chupradit, Supachai Sakkhachornphop, Sudarat Hadpech, Nattawat Onlamoon, Aftab A Ansari, Siddappa N Byrareddy, Pierre Boulanger, Saw-See Hong, Bruce E Torbett, Chatchai Tayapiwatana

**Affiliations:** 1 Division of Clinical Immunology, Department of Medical Technology, Faculty of Associated Medical Sciences, Chiang Mai University, Chiang Mai, Thailand; 2 Center of Biomolecular Therapy and Diagnostic, Faculty of Associated Medical Sciences, Chiang Mai University, Chiang Mai, Thailand; 3 Division of Clinical Immunology and Transfusion Sciences, School of Allied Health Sciences, University of Phayao, Phayao, Thailand; 4 Research Institute for Health Sciences, Chiang Mai University, Chiang Mai, Thailand; 5 Division of Instruments for Research, Office for Research and Development, Faculty of Medicine Siriraj Hospital, Mahidol University, Bangkok, Thailand; 6 Department of Pathology & Laboratory Medicine, Emory University School of Medicine, Atlanta, Georgia, USA; 7 University Lyon 1 & INRA UMR-754, Retrovirus and Comparative Pathology, Lyon, France; 8 Department of Molecular and Experimental Medicine, The Scripps Research Institute, La Jolla, California, USA

**Keywords:** anti-HIV-1 molecular scaffolds, designed ankyrin repeat proteins, designed zinc-finger proteins, HIV-1-resistant cells, SIV, SHIV

## Abstract

Designed molecular scaffolds have been proposed as alternative therapeutic agents against HIV-1. The ankyrin repeat protein (Ank^GAG^1D4) and the zinc finger protein (2LTRZFP) have recently been characterized as intracellular antivirals, but these molecules, used individually, do not completely block HIV-1 replication and propagation. The capsid-binder Ank^GAG^1D4, which inhibits HIV-1 assembly, does not prevent the genome integration of newly incoming viruses. 2LTRZFP, designed to target the 2-LTR-circle junction of HIV-1 cDNA and block HIV-1 integration, would have no antiviral effect on HIV-1-infected cells. However, simultaneous expression of these two molecules should combine the advantage of preventive and curative treatments. To test this hypothesis, the genes encoding the N-myristoylated Myr(+)Ank^GAG^1D4 protein and the 2LTRZFP were introduced into human T-cells, using a third-generation lentiviral vector. SupT1 cells stably expressing 2LTRZFP alone or with Myr(+)Ank^GAG^1D4 showed a complete resistance to HIV-1 in viral challenge. Administration of the Myr(+)Ank^GAG^1D4 vector to HIV-1-preinfected SupT1 cells resulted in a significant antiviral effect. Resistance to viral infection was also observed in primary human CD4+ T-cells stably expressing Myr(+)Ank^GAG^1D4, and challenged with HIV-1, SIVmac, or SHIV. Our data suggest that our two anti-HIV-1 molecular scaffold prototypes are promising antiviral agents for anti-HIV-1 gene therapy.

## Introduction

Human immunodeficiency virus type 1 (HIV-1) infection continues to expand worldwide, despite all the efforts put into multiple therapeutic strategies. The current standard of care for HIV-infected patients is the highly active antiretroviral therapy, which successfully reduces the HIV plasma viral load to undetectable levels and slows down AIDS progression. However, the occurrence of drug-related side effects, multidrug-resistant isolates, latent viral reservoirs, and cryptic viral replication in lymphoid tissues associated with chronic inflammation and immune dysfunction are often observed during highly active antiretroviral therapy.^1^ To date, the development of a safe and effective HIV-1 vaccine has not yet proven successful.^2^ However, recent advances in genetic manipulation of hematopoietic/progenitor stem cells, along with the development of lentiviral vector-mediated delivery of potential therapeutic genes to nondividing cells, has provided optimism for the achievement of more realistic and promising strategies for life-long protection of HIV-1-infected individuals.^3^

A number of approaches involving viral enzymatic or nonenzymatic functions have been tested as potential targets for anti-HIV-1 gene therapy. These include the intracellular expression of anti-HIV-1 agents such as shRNAs targeting the conserved long terminal repeat (LTR) region of the viral genome, or the CCR5 coreceptor, which was shown to be highly effective against R5-tropic HIV-1 replication in hu-BLT mice.^4^ U16TAR, a nucleolar-localizing TAR decoy, has been found to block the initiation of HIV-1 transcription by competing with native ligands necessary for the viral replication in human T cells.^5^ More recently, a protein-based approach has been utilized, using zinc-finger nuclease (ZFN) fusion proteins that specifically recognized the CXCR4 gene in CD4+ T-cells and CCR5 gene in hematopoietic stem cells (HSC), respectively, resulting in nonfunctional chemokine receptor cell expression, and inhibition of HIV-1 cell entry.^6,7^ Single chain variable fragment (scFv)-based strategies have also been used to negatively interfere with several strains of HIV-1, through their binding to different viral proteins, such as Tat and Gag, and to various host cell proteins including CCR5, CXCR4, and cyclin T1.^8,9,10^

However, there are severe limitations and drawbacks to these different strategies. Those involving RNA-based antivirals present an inherent lack of stability and/or specificity. In addition, due to the high mutation rates of the viral genomic RNA, the regions targeted by these agents might rapidly become insensitive to their action.^11^ Chromosome condensation can also be an obstacle, as it might obstruct the access of the ZFN to site-specific double-strand breaks in the host cell genome.^12^ In the case of scFv and intrabodies, the cytoplasm is a reducing milieu which may not favorable to the proper folding and biological function of these molecules.^13^

We have recently developed two novel intracellular protein inhibitors of HIV-1 replication, abbreviated Ank^GAG^1D4 and 2LTRZFP, as alternative antiviral molecular scaffolds for anti-HIV gene therapy. 2LTRZFP and Ank^GAG^1D4 have been designed to block HIV-1 replication at the early and late infection stages of the virus life cycle, respectively. 2LTRZFP is a zinc-finger protein (ZFP) designed to block the integration and prevent the establishment of latent viral reservoirs. 2LTRZFP is devoid of endonuclease activity, has the capacity to fold properly within the cytoplasm, and shows nuclear homing. Due to its high-affinity (in the nanomolar range) for the integrase recognition sequence at the 2-LTR circle junctions, 2LTRZFP blocks HIV-1 genome integration.^14,15^ The molecule of Ank^GAG^1D4 is an artificial ankyrin-repeat protein, which has, as do all members of the ankyrin family, a net superiority over scFv and intrabodies in terms of solubility and stability in the cytosolic reductive conditions.^16,17^ Ank^GAG^1D4 has been designed to target the HIV-1 capsid protein (CA), and its binding site is localized in the N-terminal domain of the CA (NTD^CA^). A myristoylation motif has been added to the N-terminus of Ank^GAG^1D4, resulting in the localization of Myr(+)Ank^GAG^1D4 to the plasma membrane, the site of HIV-1 assembly. Ank^GAG^1D4 has been shown to negatively interfere with viral assembly in HIV-1-infected SupT1 cells.^18,19^

The high mutation rate of the viral genome is responsible for the HIV-1 resistance to antiviral drugs. For this reason, the alteration of one single viral function is likely to be insufficient to confer full protection against chronic HIV-1 infection.^20^ Thus, the combination of multiple antiviral agents targeting two or more of the steps of the virus life cycle has a higher probability of providing a long-term protection against HIV-1 infection. The same rule applies to our anti-HIV-1 molecular scaffolds. Stable expression of Myr(+)Ank^GAG^1D4 protein in noninfected cells will not prevent the infection by new incoming viruses and the integration of their cDNA into the host-cell genome, a process which can be efficiently blocked by the 2LTRZFP scaffold, thus limiting new infection.^14,15^ On the other hand, transduction of the 2LTRZFP gene into cells already infected by HIV-1 and carrying integrated proviral genomes, is reasoned to have minimal, if any antiviral effect. In such cells however, the production and release of the viral progeny would be significantly reduced by Myr(+)Ank^GAG^1D4.^19^ Thus, the coexpression of at least two molecular scaffolds, acting at both early and late steps of the HIV-1 life cycle, would have the advantage of conferring HIV-1 resistance to heterogeneous cell populations composed of HIV-1-infected and noninfected cells. In this case, it is reasoned that whereas the application of the antiviral scaffold 2LTRZFP would serve as a preventive treatment, the Ank^GAG^1D4 would serve to control viral expression.

The aim of the present study was thus to test the validity of this hypothesis, using coexpression of 2LTRZFP and Myr(+)Ank^GAG^1D4 in human T-cells. Simultaneous expression of 2LTRZFP and Myr(+)Ank^GAG^1D4 was achieved using a constitutively expressing human T-cell line (SupT1) transduced with a third-generation lentiviral vector carrying the two transgenes, and the antiviral effect tested by challenging with HIV-1 used at high doses. The antiviral activity of Myr(+)Ank^GAG^1D4 was also evaluated in SupT1 cells preinfected with HIV-1. Simian immunodeficiency virus (SIVmac) and simian-human immunodeficiency virus (SHIV) infections in macaques are accepted as highly relevant experimental models of HIV-1 infection in humans. They have been used to study HIV-1 pathogenesis, and for preclinical testing of drugs and vaccines.^21^ We found a significant antiviral activity of Myr(+)Ank^GAG^1D4 in human primary CD4+ T-cells challenged with HIV-1, SIVmac, and SHIV. We suggest that molecular scaffolds such as designed ankyrins could be used in nonhuman primate models, and could contribute to the development of scaffold-based anti-HIV-1 gene therapy, as an alternative treatment for highly active antiretroviral therapy-resistant patients in the future.

## Results

### Construction of lentiviral vectors for constitutive, single or dual expression of anti-HIV-1 scaffolds in T-cell lines

In previous studies, we have generated stable SupT1 cell lines expressing Ank^GAG^1D4 or 2LTRZFP, using a nonintegrative, episomal vector.^14,15,18,19^ This system had the inconvenience of requiring a powerful gene delivery method and a requirement for permanent drug selection to obtain a high transduction efficiency, and a stable and reasonable level of transgene expression. This represented a major drawback for a possible application of these molecular scaffolds in stem-cell gene therapy against HIV-1. To circumvent this obstacle, we herein utilized a HIV-1-based self-inactivating (SIN) lentiviral vector to introduce anti-HIV-1 scaffold gene(s) into human CD4+ T-cells, to generate stable cell lines for constitutive expression of the transgene(s), independent of drug selection.

**Figure 1:**
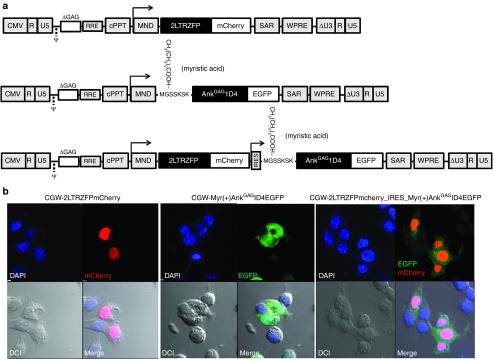
**Design of lentiviral vectors for stable transgene expression in target cells**. (**a**) Schematic diagram of self-inactivating CGW lentiviral vectors used to transfer anti-HIV-1 scaffold genes to target cells. The CGW-2LTRZFPmCherry includes the *2LTRZFP* gene in fusion with the *mCherry* reporter gene. The CGW-Myr(+)Ank^GAG^1D4EGFP vector includes the gene for *Myr(+)Ank*^*GAG*^*1D4* in fusion with the *EGFP* reporter gene. Dual vector CGW-2LTRZFPmcherry_IRES_Myr(+)Ank^GAG^1D4EGFP include both *2LTRZFP* and *Myr(+)Ank*^*GAG*^*1D4* genes in tandem, separated by an IRES. (**b**) Patterns of expression of the anti-HIV-1 molecular scaffolds in HEK293T cells. In single protein expressing cells, the red fluorescence of 2LTRZFPmCherry was found to localize within the nuclear compartment, whereas the green fluorescence of the N-myristoylated Myr(+)Ank^GAG^1D4EGFP protein was found to be associated with the intracellular membrane network and the plasma membranes. In coexpressing cells, the two proteins reached separately the cellular compartments for which they were designed, with no apparent trafficking interference. Images were taken using a Nikon C2 Plus confocal microscope, at 600× magnification. Images were analyzed using the NIS-Elements AR 4.20.00 software.

Three plasmid vectors were constructed to produce VSV-G-pseudotyped lentiviral vectors (**Figure 1a**). (i) CGW-2LTRZFPmCherry vector carried the gene coding for 2LTRZFP, fused to the mCherry reporter gene. (ii) CGW-Myr(+)Ank^GAG^1D4EGFP vector carried the Myr(+)Ank^GAG^1D4 gene, fused to the EGFP reporter gene. The enhanced green fluorescent protein (EGFP) was used as a reporter for gene expression. (iii) CGW-2LTRZFPmCherry-IRES-Myr(+)Ank^GAG^1D4EGFP vector was constructed to coexpress both anti-HIV-1 scaffolds in single-transduced cells. The gene constructs were placed under the control of the MND promoter, or MND and the IRES region (**Figure 1a**).

The levels of expression and cellular localization of Myr(+)Ank^GAG^1D4 and 2LTRZFP proteins were analyzed in HEK293T cells, using fluorescence confocal microscopy (**Figure 1b**). The red fluorescence associated with 2LTRZFPmCherry was found to localize primarily to the intranuclear region, an observation that was consistent with the nuclear homing property of ZFP, in general, and our 2LTRZFP protein in particular^14,15^ (**Figure 1b**, top and bottom leftmost panels). The green fluorescent signal of the Myr(+)Ank^GAG^1D4EGFP protein was found to localize within the cytoplasm and at the periphery of the cells (**Figure 1b**, top and bottom middle panels). This result was expected since a plasma membrane addressing signal was inserted at the N-terminus of Myr(+)Ank^GAG^1D4EGFP. Interestingly, in HEK293T cells coexpressing Myr(+)Ank^GAG^1D4EGFP and 2LTRZFPmCherry, the two molecules trafficked independent of their preferred cellular compartments (**Figure 1b**, top and bottom rightmost panels). Of note, the percentages of fluorescent signal-positive cells were 80, 90, and 60%, respectively (data not shown). This result indicated that the SIN lentiviral vectors represented an efficient vector for the delivery of *Myr(+)Ank*^*GAG*^*1D4EGFP* and *2LTRZFPmCherry* genes along with, high-level expression of the antiviral scaffolds in target cells.

The production of VSV-G-pseudotyped lentiviral vectors carrying the gene for 2LTRZFPmCherry, Myr(+)Ank^GAG^1D4EGFP, or both anti-HIV scaffolds, was also performed in HEK293T cells cotransduced with vectors (i), (ii), or (iii), along with two additional vectors required for genome packaging and particle production (pMDLgag/polRRE and pRSV-Rev), and a third vector for VSV-G pseudotyping (pMD.2G).^10^

### Antiviral activity in anti-HIV-1 scaffold-expressing SupT1 cells

**Figure 2:**
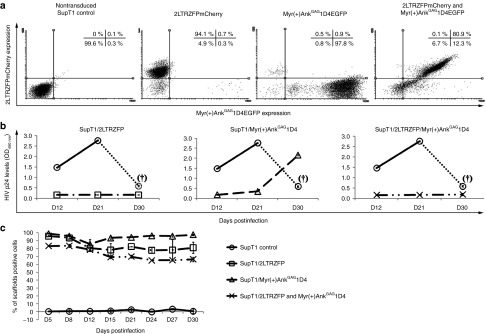
**Inhibition of viral replication in single and double anti-HIV-1 scaffold-expressing cells**. (**a**) Flow cytometry analysis. Control, nontransduced SupT1 cells and CGW-transduced SupT1 stably expressing anti-HIV-1 scaffolds were harvested at day 30 post-transduction, and the expression profiles of 2LTRZFPmCherry, Myr(+)Ank^GAG^1D4EGFP, or both analyzed by flow cytometry. The frequencies of events within each quadrant are shown with each profile. (**b**) HIV-1 challenge. Aliquots of nontransduced SupT1 cells and SupT1 cells stably expressing 2LTRZFP (leftmost panel), Myr(+)Ank^GAG^1D4 and (middle panel), or the combination of 2LTRZFP and Myr(+)Ank^GAG^1D4 (rightmost panel) were infected with HIV-1_NL4-3_ at multiplicity of infection (MOI) 20. Culture supernatants collected on day 12, 21, and 30 postinfection (pi) were analyzed by enzyme-linked immunosorbent assay for extracellular p24 antigen. Note that in control SupT1 cells, >90% cells were dead at day 30 (dotted line and symbol (†)). (**c**) Expression of mCherry and EGFP in HIV-1-infected cells. Aliquots of CGW-transduced, HIV-1- challenged SupT1 cells were collected at different times pi, and analyzed by flow cytometry. The data presented were the average from triplicate experiments. Bars represent mean ± SEM.

Our next experiments were designed to evaluate the level of protection against HIV-1 of CD4+ T-cells expressing Myr(+)Ank^GAG^1D4 alone, 2LTRZFP alone, or the two molecular scaffolds together. The human T-lymphocytic cell line SupT1, transduced by the lentiviral vectors described above, were maintained in culture for 30 days before HIV-1 infection. Highly enriched population of cells expressing either EGFP-fused Myr(+)Ank^GAG^1D4 alone, mCherry-fused 2LTRZFP alone, or both were isolated by flow cytometry cell sorting. Cells positive for EGFP or mCherry (≥ 80% at day-30 post-transduction; D30 pt) were challenged with HIV-1 at a multiplicity of infection (MOI) of 20; (**Figure 2a**), and the degree of antiviral protection was evaluated in culture supernatants collected at D12, D21, and D30 postinfection (pi) using a p24 enzyme-linked immunosorbent assay (ELISA) (**Figure 2b**). The monitoring of HIV-1-infected cells for the stability of expression of the antiviral molecular scaffolds showed that >80% of the cells were positive for EGFP or mCherry at D12 pi, and between 70 and 95% cells remain positive at D30 pi (**Figure 2c**).

**Figure 3:**
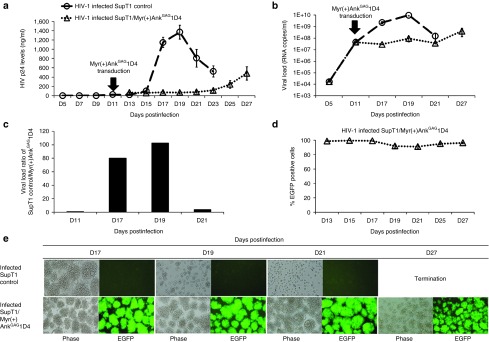
**Antiviral effect of Myr(+)Ank**^**GAG**^**1D4 scaffold on HIV-1-infected cells**. (**a–c**) Viral production. SupT1 cells infected with HIV-1_NL4-3_ at multiplicity of infection (MOI) 1 were collected on day 11 pi, and divided into two aliquots, one transduced with CGW-Myr(+)Ank^GAG^1D4EGFP vector at MOI 0.7, the other serving as control. The cell culture supernatants were collected at 3-day intervals, from day 5 to day 27 post-transduction, and assayed for p24 antigen (**a**) and viral load (**b**). The fold changes in viral loads between control and CGW-Myr(+)Ank^GAG^1D4EGFP-transduced SupT1 cells were calculated for days 11, 17, 19, and 21, and are shown in (**c**). The data presented were the average (mean) from triplicate experiments (mean ± SEM). (**d,e**) Flow cytometry and microscopy. The level of EGFP expression over the time of infection was evaluated by standard flow cytometry (**d**). The cell morphology and EGFP expression were monitored by light and fluorescence microscopy, using an inverted fluorescence microscope at 200× magnification (**e**).

In control SupT1 cells as expected, the supernatant fluids collected on D12 and D21 from the nontransduced cells infected with HIV-1 showed high p24 levels. The decrease in supernatant fluids collected from these cultures after D21 was due to the high cell mortality (90% nonviable cells) at D21 (refer to **Figure 3e**). In contrast, the level of p24 antigen was undetectable in supernatant fluids collected from HIV-1 infected cultures of cells transduced with the CGW vectors carrying the genes for Myr(+)Ank^GAG^1D4EGFP, 2LTRZFPmCherry, or both, until D21 pi. However, levels of p24 antigen increase in supernatant fluids collected between D21 and D30 in the SupT1/Myr(+)Ank^GAG^1D4 (single expression), but remained undetectable in SupT1/2LTRZFP (single expression) and SupT1/2LTRZFP/Myr(+)Ank^GAG^1D4 transduced cells (dual expression; **Figure 2b**).

These findings confirmed our starting hypothesis that an efficient and long-term protection against HIV-1 infection at high MOI could not be conferred by one single type of molecular scaffold, like those targeting the late steps of the virus life cycle (Myr(+)Ank^GAG^1D4), but also required the blockage of another step of the virus life cycle. This could be achieved by 2LTRZFP, which was found to efficiently inhibit the viral integration, even in SupT1 cells infected with a high dose of HIV-1, as shown by *Alu-gag* quantitative real-time polymerase chain reaction (PCR) (*Alu-gag* qPCR; **Supplementary Table S1**).

### Myr(+)Ank^GAG^1D4-mediated antiviral effect in HIV-1-preinfected SupT1 cells

Due to the complete inhibition of viral integration and replication observed with 2LTRZFP (**Figure 2b** and **Supplementary Table S1**), there was no virus escape which would allow us to evaluate the possible anti-HIV-1 function of Myr(+)Ank^GAG^1D4 at the postintegration steps. Myr(+)Ank^GAG^1D4 would theoretically block the virus assembly process in HIV-1-infected cells. Therefore, we used SupT1 cells preinfected with HIV-1 as the cellular model to measure the contribution of Myr(+)Ank^GAG^1D4 to the global antiviral effect produced by the combined vectors.

We next tested the antiviral effect of Myr(+)Ank^GAG^1D4 in SupT1 cells preinfected with HIV-1 at low MOI 1, in order to mimic the scenario of chronically produced virus. The status of HIV-1-infected cells was verified by p24 ELISA and levels of virus in the supernatant fluids using standard RT-PCR assays. Supernatant fluids collected on day 11 postinfection gave p24 values of 25 ng/ml and 4.5 × 10^7^ genomic RNA copies/ml for the viral load (**Figure 3a**,**b**).

HIV-1-infected SupT1 cells were then harvested on D11 and while one aliquot was transduced with the CGW lentivector carrying the gene for Myr(+)Ank^GAG^1D4, the other aliquot was nontransduced and served as control (**Figure 3a**). Following HIV-1 infection, low levels of p24 antigen were observed in the supernatants fluids from the SupT1/Myr(+)Ank^GAG^1D4 (≤ 50 ng/ml) until D21 postinfection, *i.e.*, 10 days after vector transduction (**Figure 3a**). This result was confirmed by the viral load assays, which showed a 80- and 100-fold reduction in samples collected on D17 and D19 postinfection, respectively as compared to nontransduced HIV-1-infected cells (**Figure 3b**,**c**). The levels of ankyrin expression in SupT1/Myr(+)Ank^GAG^1D4 cells were found to be very high until D27 postinfection (16 days after vector transduction), at 90% EGFP-positive cells (**Figure 3d**,**e**). In control, nontransduced cells, a peak of extracellular p24 antigen was observed in supernatant fluids collection on D19, with a decrease in p24 occurring after D21, due to cell apoptosis induced by HIV-1 (**Figure 3e**).

The residual levels of extracellular p24 antigen after D21 raised the issue of the occurrence of viral mutants which would escape the Myr(+)Ank^GAG^1D4-mediated inhibition of HIV-1 replication. In our previous report, we characterized the key amino acids in the HIV-1 capsid protein that play the important role in the Ank^GAG^1D4-NTD^CA^ interaction. The arginine-18 (R18) in helix 1, and R132 and R143 in helix 7 are the key players of this interaction.^22^ We have aligned the amino acid sequences of helix 1 and helix 7 of the HIV-1 subtype B prototype with newly isolated strains, deposited in the HIV sequence database from 2008 to 2013. We found that R18 in helix 1, and R132 and R143 in helix 7, are highly conserved residues in the CA sequence of all isolates (not shown). These amino acid residues are known to be crucial for the morphogenesis of the virions and their stability, and the chances that mutations of these amino acids would result in viable viruses are minimal. However, the possibility of a viral escape from the Myr(+)Ank^GAG^1D4 antiviral activity should be only definitely eliminated by challenging Myr(+)Ank^GAG^1D4-expressing cells with various HIV-1 CA mutants.

The explanation to the persistence of p24 antigen in the extracellular medium of HIV-1-infected SupT1/Myr(+)Ank^GAG^1D4 cells more likely resided in the intrinsic properties of Myr(+)Ank^GAG^1D4. Myr(+)Ank^GAG^1D4 has been found to bind to the HIV-1 capsid with a moderate affinity (*K*_d_ ~ 1 μmol/l).^19^ Thus, Myr(+)Ank^GAG^1D4-expressing cells would still allow some HIV-1 to leak out in the extracellular milieu. This difficulty can be overcome in future applications of therapeutic ankyrins designed to inhibit the HIV-1 assembly. It will be possible to significantly enhance the affinity of Myr(+)Ank^GAG^1D4 to the HIV-1 capsid by site-directed mutagenesis of important amino acid residues of the binding site of the ankyrin protein to increase its binding affinity to its viral target, as described by Sammond *et al*.^23^

### Antiviral protection of primary CD4+ T-cells with heterogeneous Myr(+)Ank^GAG^1D4 expression

Autologous gene-modified CD4+ T-cells in HIV-1-infected patient have been identified as one of promising alternative HIV treatments.^24^ In this strategy, CD4+ T-cells from a HIV-1-infected individual are isolated, anti-HIV-1 genes introduced, and cells expanded *ex vivo* before reinfusing them back into the body. However, the expanded CD4+T-cell population might contain cells of the HIV latent reservoir that would be ready to rereplicate after infusion.^1^ As a consequence, it is essential to select therapeutic gene(s) which are able to control the expression of latent HIV-infected cells. In this context, the application of Myr(+)Ank^GAG^1D4 to autologous adoptive T-cell transfer would have the advantage of preventing viral replication from both preinfected and naive cells.

It was important to model *in vitro* the degree of antiviral protection of a human primary CD4+ T-cell population, which, in individuals subjected to gene therapy, would be heterogeneous in terms of antiviral scaffold expression. The *in vivo* distribution of therapeutic vector among the CD4+ T-cell population would not be homogeneous, with transgene expression in certain cells, but not in others, and scaffold expression would occur at various levels. Therefore, an experimental setup was designed to mimic this type of *in vivo* heterogeneity, and to study its influence on the cell susceptibility to HIV-1.

**Figure 4:**
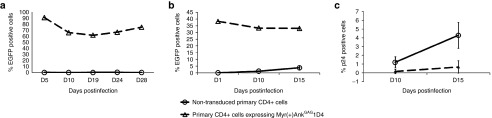
**Myr(+)Ank**^**GAG**^**1D4-mediated protection of primary CD4+ T-cells against HIV-1 challenge**. Human primary CD4+ T-cells were purified from whole-blood samples obtained from healthy donors, and transduced with the CGW-Myr(+)Ank^GAG^1D4EGFP lentiviral vector at multiplicity of infection (MOI) 4. (**a**) The Myr(+)Ank^GAG^1D4EGFP expression was found to be stable over the time, with a plateau at approximately 70% EGFP-positive cells, as monitored by flow cytometry. The percentage of Myr(+)Ank^GAG^1D4EGFP-positive cells was then adjusted to approximately 30% by dilution with nontransduced primary CD4+ T-cells, and the mixed T-cell population was infected with X4-tropic HIV-1 at high MOI (500ng of HIV-1_NL4-3_ p24 per 10^6^ cells). (**b**) The stability of Myr(+)Ank^GAG^1D4EGFP expression over the time of HIV-1 infection was monitored by standard flow cytometry. (**c**) Aliquots of the HIV-1-infected, mixed cell population were collected at day 10 and day 15 pi, and the percentage of HIV-1-infected cells determined by immunostaining of intracellular p24 antigen. The data presented were the average (mean) from triplicate experiments (mean ± SEM).

Replicates cultures of primary CD4+ T-cells were stimulated *in vitro* with anti-CD3/CD28 antibodies, a treatment which is reported to downregulate CCR5 receptors at the cell surface, and results in a high-level resistance to R5-tropic virus.^25,26^ Anti-CD3/CD28-activated CD4+ T-cells were then transduced with the CGW-Myr(+)Ank^GAG^1D4EGFP vector. The expanding population showed at least 95% cell viability and stably expressed Myr(+)Ank^GAG^1D4EGFP protein over a long time period (up to D28), with about 70% EGFP-positive cells, as monitored by flow cytometry (**Figure 4a**). The 70% EGFP-positive cell culture was diluted with non-transduced primary anti-CD3/CD28 activated CD4+ T-cells, to obtain a final value of 30% EGFP-positive cells. The mixed population of Myr(+)Ank^GAG^1D4-positive (30%) and Myr(+)Ank^GAG^1D4-negative (70%) CD4+ T-cells was then challenged with X4-tropic HIV-1 (HIV-1_NL4-3_) at MOI of 500 ng of p24 per 10^6^ cells (corresponding to a MOI of 20). Controls consisted of nontransduced primary CD4+ T-cells infected with the same dose of HIV-1_NL4-3_. We verified that the percentage of Myr(+)Ank^GAG^1D4EGFP-positive cells remained stable throughout the virus challenge, at approximately the same value as at time 0 of the experiment (30%; **Figure 4b**). Cells were collected at D10 and D15 postinfection, and the number of HIV-1-infected cells was determined by intracellular p24 protein assessment by flow cytometry. As shown in **Figure 4c**, the expression of Myr(+)Ank^GAG^1D4 in only 30% human primary CD4+ T-cells provided a significant protection against X4-tropic HIV-1, with a 7.2-fold lower number of HIV-1-positive cells, as compared to controls.

### Ank^GAG^1D4-mediated antiviral activity against SIVmac and SHIV

We then examined the capability of Myr(+)Ank^GAG^1D4 to block the simian immunodeficiency virus (SIV) and chimeric simian-human immunodeficiency virus (SHIV) infection *in vitro*. It has been shown that human T-lymphocyte cells SupT1 can support SIV and SHIV replication.^27^ Therefore, this cell line could be used as the host cells to test the ankyrin function. Moreover, since the MND promoter is able to drive the expression of transgenes in both human and macaque cells with the same efficiency,^28^ the levels of expression of ankyrins in human-derived cells would be similar to those which would occur in macaque-derived cells.

**Figure 5:**
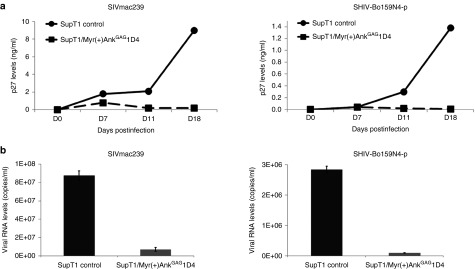
**Myr(+)Ank**^**GAG**^**1D4-mediated inhibition of SIVmac and SHIV replication in SupT1 cells**. (**a**) SupT1 cells stably expressing Myr(+)Ank^GAG^1D4EGFP were infected with SIVmac239 or SHIV-Bo159N4-p at multiplicity of infection of 1. Culture supernatants were collected at days 7, 11, and 18 pi, and the levels of p27 antigen determined by enzyme-linked immunosorbent assay. (**b**) The numbers of viral genome copies were evaluated by qPCR in the cell culture supernatants collected on day 18 pi.

SupT1 cells stably expressing Myr(+)Ank^GAG^1D4EGFP were infected with SIVmac239 or SHIV-Bo159N4-p at the same doses (MOI of 1; **Figure 5**). Cell culture supernatants were collected at D7, D11, and D18 postinfection. The concentration of extracellular p27 antigen was determined by ELISA, and that of viral genomes determined by qPCR. EGFP expression was verified by flow cytometry at D18 postinfection (**Supplementary Figure S1**). As shown by ELISA, the p27 levels were significantly decreased at D11 and D18 in both SIV and SHIV challenges, compared to control SupT1 cells (**Figure 5a**). Likewise, viral genomes were almost undetectable in the culture supernatant of Myr(+)Ank^GAG^1D4EGFP-expressing cells at D18 (**Figure 5b**). These results indicated that Myr(+)Ank^GAG^1D4 possessed a broad antiviral activity against HIV-1, SIV, and SHIV. Of note, flow cytometry analysis for the expression of the Myr(+)Ank^GAG^1D4 showed that a high frequency of the SIV- and SHIV-infected cells expressed EGFP (**Supplementary Figure S1**).

This broad antiviral activity was not surprising, considering the CA sequence homology between HIV-1, SIVmac and SHIV, and the findings of our recent studies. We found that Ank^GAG^1D4 specifically interacted with helix 1 and helix 7 of NTD^CA^, the N-terminal domain of HIV-1 capsid protein.^22^ Helices 1 of HIV-1 and SIVmac239 NTD^CA^ have 93.8% sequence homology, and 75% sequence identity, and helices 7 have 89.5% homology and 73.7% identity (**Supplementary Figure S2**). Ank^GAG^1D4 therefore negatively interfered with the assembly process of SIV and SHIV as efficiently as with that of HIV-1 virus particles. This is in contrast with the 2LTR circle junction sequences of SIV and SHIV, which significantly differ from that of HIV, with only 59.1% homology.^29,30^ As a result of this heterogeneity, the 2LTRZFP protein would probably not bind to the 2LTR circle junctions of SIV and SHIV, or bind to them with a very low affinity. Thus, only the antiviral activity of Myr(+)Ank^GAG^1D4 against with SIV and SHIV was evaluated in this experiment.

## Discussion

Various proteins with anti-HIV-1 activity have been designed in recent years. This includes a zinc-finger antiviral protein, which promotes the specific degradation of viral mRNAs and has been shown to inhibit HIV-1 infection.^31^ However, the occurrence of escape mutation(s) in the zinc-finger antiviral protein-targeted region(s) of the mRNAs remains an important concern. Likewise, a CD4-specific designed ankyrin-repeat protein (DARPin), which binds to the N-terminal immunoglobulin-like domain of human CD4, inhibits the entry of various HIV-1 strains.^32^ However, the high rate of blood clearance of this CD4-specific DARPin is still an obstacle to potential clinical applications.^33^ A gp120-specific DARPin, which recognizes the V3 loop of HIV-1 gp120 was found to function as a viral entry inhibitor,^34^ but the high mutation rate of gp120 represents a major challenge for its use in antiviral gene therapy, as well as for vaccine and drug development.^35^ In addition, zinc finger nuclease*s* (ZFNs) that target human CCR5 and CXCR4 receptors also inhibit the cellular internalization of both R5- and X4-tropic HIV-1.^36^ However, disruption of chemokine receptors could negatively affect immune cell function, and induce chronic inflammation.^37,38^ The antiviral activity of ZFNU3, a zinc-finger nuclease which excises the proviral DNA from the host chromosome,^39^ might be limited by the poor accessibility of the proviral DNA integrated into condensed regions of the chromosome.^12^

By comparison with these different types of anti-HIV-1 proteins and their limitations, HIV-1-targeted intracellular molecular scaffolds, such as the designed ZFPs and ankyrin-repeat proteins defined herein, could offer an alternative and a more reasonable approach for antiviral gene therapy. Their multiple advantages include their expression stability, absence of cytotoxicity, and the potential to manipulate their active site and binding affinity with their ligands, as well as the possibility to modify their intracellular trafficking to the most appropriate host cell compartment(s), for example via the addition of specific compartment-addressing signals, such as N-myristoylation or nuclear localization signal. The two molecular scaffolds which was the focus of our present study, 2LTRZFP and Myr(+)Ank^GAG^1D4, both exerted their intracellular antiviral activity towards conserved structural elements of the HIV-1 virion, or critical viral functions which take place in specific host-cell compartments. The 2LTRZFP construct possesses nuclear homing property and has been shown to inhibit the integration of HIV-1 provirus.^14,15^ Ank^GAG^1D4, in its N-myristoylated version Myr(+)Ank^GAG^1D4, was directed to the plasma membrane where it negatively interfered with the oligomerization of the Gag proteins and the assembly of HIV-1 capsid.^18,19^

The genes for both the molecular scaffolds 2LTRZFP and Myr(+)Ank^GAG^1D4 were transduced into SupT1 cells or human primary CD4+ T-cells, using a single HIV-1-based, SIN CGW lentiviral vector. Plasmids for genome vector packaging and VSV-G-pseudotyping were provided in *trans*. The vector genome carried a scaffold attachment region element, to increase the transgene product expression and provide protection against gene silencing in human lymphoid and myeloid cells.^40^ The transgenes were placed under the control of the MND promoter, to enhance the expression of therapeutic genes in hematopoietic cells, including primary CD4+ T-cells and hematopoietic stem cells.^41^ We found that both molecular scaffolds were stably expressed in human T-cells. Interestingly, the simultaneously expressed 2LTRZFP and Myr(+)Ank^GAG^1D4 proteins showed independent trafficking to their respective homing compartments, the nucleus for 2LTRZFP, and the plasma membrane for Myr(+)Ank^GAG^1D4. We submit that SIN CGW lentiviral vectors are efficacious tools for multiple transgenes delivery and for the stable expression of the transgene products in human T-cells.

Considering the problems of anti-HIV-1 drug adherence, viral-resistant strains, and latent viral reservoirs, new therapeutic approaches using anti-HIV-1 gene therapies combined with HSC-based therapies have emerged as a promising direction for curing HIV-1-infected individuals with a single-time treatment. HSCs are capable for self-renewal and differentiation into multilineage hematopoietic cell types, including T-lymphocytes and macrophages that have been shown to serve as the primary targets of HIV-1. Hence, transplantation of modified HSCs containing anti-HIV-1 genes in AIDS patient could produce new immune cells that would resist HIV-1 infection. Autologous hematopoietic stem cell transplantation is an alternative source of HSCs without the potential complications associated with graft-versus-host disease.^42^ However, there are many reports that CD34+ progenitor cells derived from granulocyte-colony stimulating factor (G-CSF)-mobilized peripheral blood or bone marrow cells are susceptible to HIV-1 infection.^43,44^ Thus, autologous CD34+ cell transplantation of HIV-1-infected patients might introduce, as the Trojan horse, cellular reservoirs of HIV-1 with the risk of virus spread to various anatomic compartments.^44^ It therefore seems logical to consider the modification of autologous CD34+ progenitor cells by appropriate antiviral genes, to render them safe for transplantation, while preserving the essential cellular functions.

Our molecular scaffolds, 2LTRZFP and Myr(+)Ank^GAG^1D4 proteins, fulfilled the requirements for stem cell-based gene therapy against HIV-1 infection. When transduced in tandem by a single lentiviral vector of the SIN-CGW type, the two scaffolds will provide both preventive effects and control of viral production. (i) 2LTRZFP, which blocks the early step of viral integration step, will protect noninfected cells from infection by new incoming viruses, although it would have no effect on HIV-1-infected cells already carrying integrated provirus, and (ii) Myr(+)Ank^GAG^1D4 protein, which cannot protect noninfected cells against the viral integration of newly incoming HIV-1, can exert its antiviral effect on HIV-1-infected cells, by inhibiting the late step of assembly and release of progeny virions. Hence, transplantation of HIV-1-infected individuals with autologous HSCs modified to express both 2LTRZFP and Myr(+)Ank^GAG^1D4 proteins, would confer robust antiviral immunity to the recipients, not only via the production of HIV-resistant cells, but any cell that was infected would be actively controlled and no virus would be produced.

Experiments in nonhuman primate models will be necessary to further characterize the biosafety and antiviral activity of our molecular scaffolds. In the present study, we found that the Myr(+)Ank^GAG^1D4 protein expressed in human SupT1 cells was a strong inhibitor of SIVmac and SHIV replication. Collectively, our data suggests that our two molecular scaffolds 2LTRZFP and Ank^GAG^1D4, coexpressed in target cells using a unique lentiviral vector carrying both genes in tandem, represented an novel class of intracellular antiretrovirals with interesting therapeutic potential, most notably in future hematopoietic stem cell-based anti-HIV-1 therapy.

## Materials and methods

*Cells*

*Human cell lines*. HEK293T (human embryonic kidney 293T) and SupT1 cells (a human T-lymphoid cell line susceptible to HIV-1 infection) were obtained from the ATCC (Manassas, VA).^6,10,14,19^ HEK293T cells were maintained in Dulbecco's modified Eagle's medium (Gibco, Grand Island, NY) and SupT1 cells were grown in RPMI-1640 medium, both supplemented with penicillin (100 U/ml), streptomycin (100 µg/ml), L-glutamine (2 mmol/l), and 10% fetal bovine serum (Hyclone, Cramlington, UK). All cell cultures were maintained in a 37 °C humidified incubator containing 5% CO_2_.

*Human primary CD4+ T-cells*. Whole-blood samples were obtained from healthy donors at the Normal Blood Donation Center after approval by The Scripps Research Institute Institutional Review Board, La Jolla, CA. peripheral blood mononuclear cells (PBMCs) were isolated from peripheral blood by density-gradient centrifugation.^6,10^ Isolation of highly pure CD4+ T-cells was achieved by using EasySep Human CD4+ T-Cell Enrichment Kit (Stem Cell Technologies, Vancouver, Canada). Determination of purity of over 95% was determined using flow cytometry with appropriate reagents. Primary CD4+ T-cells were maintained in RPMI-1640 medium, supplemented with 10% fetal bovine serum (Hyclone, Cramlington, UK), interleukin-2 (IL-2) (100 U/ml) (Peprotech, Rocky Hill, NJ), L-glutamine (2 mmol/l), nonessential amino acids (NEAA) (0.1 mmol/l), sodium pyruvate (1 mmol/l), penicillin (100 U/ml), and streptomycin (100 µg/ml). They were stimulated with anti-CD3/CD28-conjugated Dynabeads (human T-activator, Invitrogen, Carlsbad, CA), and reactivated with the anti-CD3/CD28 beads and IL-2 every 5 days during cell culture.

*Construction of lentiviral vectors CGW,^10^ a third-generation lentiviral vector, was used as the backbone vector to transfer the genes for our molecular scaffolds 2LTRZFP and Myr(+)Ank^GAG^1D4 into target cells*. This transfer vector carried three unique elements: (i) the MND promoter (a synthetic promoter that contains the U3 region of a modified MoMuLV LTR with myeloproliferative sarcoma virus enhancer), to ensure a high expression of the gene of interest;^10^ (ii) β-interferon (IFN) scaffold attachment region element, which has been shown to improve transgene expression by inhibiting gene methylation and promote protection from gene silencing in both resting and activated hematopoietic cells, including human T and myeloid cells;^40^ and (iii) cellular internal ribosomal entry site (cIRES), to control the bicistronic transgene production^10^ and allow for a cap-independent translation of both 2LTRZFP and Myr(+)Ank^GAG^1D4 proteins in the same target cell.

*CGW-2LTRZFPmCherry*. The fragment containing the *2LTRZFP* gene was excised from the p156mPGK-ZFP-GFP vector,^14^ using *Xba* I and *Xma* I, and inserted into pUC18. The 2LTRZFP-containing fragment was rescued by excising with *EcoR* I and *BstX* I, and reinserted into the *EcoR* I and *BstX* I sites of the CGW lentiviral vector. The resulting fusion protein, 2LTRZFPmCherry, possessed the mCherry fluorescent protein at its C-terminus.

*CGW-Myr(+)Ank^GAG^1D4EGFP*. The *Ank*^*GAG*^*1D4* gene-containing DNA fragment was rescued by PCR amplification from the pCEP4-Myr(+)Ank^GAG^1D4GFP plasmid.^19^ The PCR fragment was cloned into the CGW vector linearized by digestion with *EcoR* I and *BstX* I. The fusion protein Myr(+)Ank^GAG^1D4GFP carried a N-myristoylation signal at its N-terminus for targeting to the plasma membrane, and the in-phase EGFP sequence at its C-terminus.

*CGW-2LTRZFPmCherry_IRES_Myr(+)Ank^GAG^1D4EGFP*. This bicistronic gene-carrying vector was generated to coexpress both 2LTRZFPmCherry and Myr(+)Ank^GAG^1D4EGFP proteins in a single cell. The DNA fragment containing the *2LTRZFPmCherry* gene was excised from the CGW-2LTRZFPmCherry vector treated with *EcoR* I and *BsrG* I, and reinserted into the CGW-Myr(+)Ank^GAG^1D4EGFP vector, upstream to the IRES element. In the resulting transfer vector, the *2LTRZFPmCherry* gene was positioned downstream to the MND promoter, and the *Myr(+)Ank*^*GAG*^*1D4EGFP* gene was placed downstream to the IRES element.

*Production of VSV-G-pseudotyped lentiviral vectors*. VSV-G-pseudotyped lentiviral vector particles were produced in HEK293T cells, using four separate plasmids and the calcium phosphate cotransfection method, as previously described.^10,45^ HEK293T cells were seeded on 10-cm dishes (3.5 × 10^6^ cells per dish), and cotransfected with the CGW transfer vector (10 µg/dish), the packaging construct pMDLg/pRRE (6.5 µg/dish), pRSV-Rev (2.5 µg/dish), and pMD.2G (3.5 µg/dish). Vector particles were harvested from the culture supernatant collected at 24 and 48 hours, and high-titer viral vector stocks prepared by ultracentrifugation.^10,45^ The viral vector titers, determined by infection of HEK293T cells with serial dilution of the samples, were expressed as the percentage of EGFP- or mCherry-positive cells.^45^

*Generation of cells stably expressing 2LTRZFPmCherry and Myr(+)Ank^GAG^1D4EGFP*. SupT1 and HEK293T cells were transduced with the VSV-G-pseudotyped CGW vectors at the MOI of 1. Primary CD4+ T-cells were activated with anti-CD3/CD28 beads in media containing 100 U/ml IL-2 for 24 hours, and transduced with CGW-Myr(+)Ank^GAG^1D4EGFP at MOI 4. For the transduction of HIV-infected cells, CGW-Myr(+)Ank^GAG^1D4EGFP was added to HIV-infected SupT1 cells at MOI 0.7, subjected to spinoculation by centrifugation at 2,000×*g* at 32 °C for 1.5 hours, in a growth medium containing 8 µg/ml of Polybrene (Sigma–Aldrich, St. Louis, MO). The cells were then transferred to a humidified incubator, and maintained at 37 °C and 5% CO_2_ for 24 hours. The cells were then washed three times with fresh growth medium and further cultured in fresh growth medium and divided every 5 days for the primary T-cells or every 3 days for the cell lines. The efficiency and stability of transduction was determined by fluorescence microscopy and flow cytometry (FACSCalibur; BD Biosciences, Le Pont de Claix, France). SupT1 cells that expressed antiviral molecular scaffolds were isolated using a FACS sorter (FACSAria III sorter, BD Biosciences).

*Confocal microscopy*. HEK293T cells transduced with each VSV-G-pseudotype lentiviral vector at MOI 1 were seeded on cover glass slides, and left overnight for culturing. Cells were fixed with 4% formaldehyde in PBS, and permeabilized with 0.2% Triton X-100. Then, the nucleus was counterstained with DAPI. Images were acquired using Nikon C2 Plus confocal microscope with 600× magnification, and analyzed using the NIS-Elements AR 4.20.00 software.

*Viral stocks*. Replication-competent HIV-1_NL4-3_ virus (X4-tropic) was produced by transient transfection of pNL4-3 plasmid into HEK293T cells. Monolayers of HEK293T cells (3.5 × 10^6^ cells per 10-cm dishes) were transfected with 5 µg of the pNL4-3 plasmid, using Lipofectamine and Plus reagents (Life Technologies, Carlsbad, CA), as previously described.^14^ After 5 hours, the transfection mixture was withdrawn, replaced by 10 ml of growth medium, and the cells were allowed to grow for 48 hours. HIV-1 virus was harvested from the culture supernatants, by filtration through sterile syringe filter*s* with a 0.45-µm pore size (Millex-HA filter unit; Merck Millipore, Hessen, Germany). HIV-1 samples were aliquoted and kept frozen at −80 °C. The virus titer was determined using conventional p24 antigen ELISA, using the Genscreen ULTRA HIV Ag-Ab assay (Bio-Rad, Marnes-la-Coquette, France). The viral load was determined using the COBAS AMPLICOR HIV-1 Monitor test (version 1.5; Roche Molecular Systems, Branchburg, NJ).

Viral stocks of SIVmac239 (ref. ^46^) and SHIV-Bo159N4-p (^47^) were produced by infection of Concanavalin A-stimulated naïve rhesus macaque PBMCs, and infected cell cultures were maintained in the presence of IL-2 (20 U/ml), as previously described.^48^

*HIV-1 infection*. The level of antiviral protection conferred by our molecular scaffolds was evaluated by challenge with the X4-tropic HIV-1_NL4-3_. Control SupT1 cells and SupT1 cells stably expressing 2LTRZFP, Myr(+)Ank^GAG^1D4 or both, were maintained in growth medium for at least 4 weeks before HIV-1 infection. The cells were incubated with HIV-1, added at MOI 20, for 16 hours. The cells were then washed three times with serum-free medium, and resuspended in fresh growth medium. They were split (1:2) at 3-day intervals, to maintain a cell density of approximately 10^6^ cells/ml. HIV-1 replication was monitored in culture supernatants, using p24 antigen ELISA and viral load assay, as described above. The cell pellets were kept to determine the level of inhibition of proviral integration. The cell viability was assessed by the trypan blue dye exclusion staining method. For viral challenges, the SupT1 cells were infected with HIV-1_NL4-3_ at MOI 1, using the method described above. Then, on day 11 postinfection (pi), cells were transduced by the CGW-Myr(+)Ank^GAG^1D4EGFP lentiviral vector at MOI 0.7, as described above.

For infection of primary CD4+ T-cells, the percentage of Myr(+)Ank^GAG^1D4EGFP-positive cells was adjusted to approximately 30% by the addition of nontransduced cells, and the mixed cell cultures were infected with 500 ng of HIV-1_NL4-3_ p24 per 10^6^ cells. Aliquots of HIV-1-infected cells were collected at 5-day intervals, and the progression of HIV-1 replication was detected by immunofluorescent staining of intracellular p24 protein and flow cytometry. The cells were fixed and permeabilized using BD Cytofix/Cytoperm Fixation/Permeabilization Solution Kit (BD Biosciences, San Diego, CA), and incubated for 30 minutes with PE-conjugated anti-p24 antibody (clone KC57-RD1; Beckman Coulter, Fullerton, CA). Cells were incubated with control IgG isotype antibodies to evaluate the level of nonspecific binding. Stained cells were then analyzed using the BD FACS Calibur LSR II flow cytometer, using the CELLQUEST software.

*HIV-1 integration assay*. The number of viral genome copies integrated into the host DNA of control SupT1 and SupT1 cells stably expressing molecular scaffolds was determined by using a conventional *Alu-gag* qPCR assay, as described previously.^49,50^ Briefly, the DNA of HIV-1-infected control SupT1 and CGW-vector-transduced SupT1 cells was extracted (High Pure PCR template kit; Roche, Mannheim, Germany), and a first round of PCR performed using a pair of primers specific for the *Alu* (human) and *gag* (HIV-1) sequences. The primers for the first-round amplification were the following: *Alu* forward, 5′-GCC TCC CAA AGT GCT GGG ATT ACA G-3′, and HIV-1 *gag* reverse, 5′-GTT CCT GCT ATG TCA CTT CC-3′. The reactions were performed in a total reaction volume of 25 μl (5 PRIME, Gaithersburg, MD), using a standard protocol.^14^ The second-round of RU5 kinetic PCR was performed on 10 μl of diluted (1:4) first-round PCR product. The primer sequences were R_FWD, 5′-TTA AGC CTC AAT AAA GCT TGC C-3′ and U5 _REV, 5′-GTT CGG GCG CCA CTG CTA GA-3′. The RU5 molecular beacon probe, which was labeled at its 5′ terminus with the 6-carboxyfluorescein (FAM), as the reporter dye and at its 3′ terminus with the BlackBerry quencher (BBQ), had the following sequence: 5′-FAM-CCA GAG TCA CAC AAC AGA CGG GCA CABBQ-3′. The reactions were performed in a final volume of 25 µl containing DyNAmo probe qPCR master mix (Finnzymes, Espoo, Finland), 400 nmol/l RU5 (R_FWD) primer, 400 nmol/l RU5 (U5_REV) primer, and 140 nmol/l RU5 molecular beacon probe. The reactions were performed using the CFX96 real-time PCR system with the following program: 20-second hot start at 95 °C, followed by 50 cycles of denaturation at 95 °C for 3 seconds and annealing and extension at 63 °C for 30 seconds. Glyceraldehyde-3-phosphate dehydrogenase (GAPDH) was used for quantifying the amount of DNA in each qPCR assay, by using the following GAPDH primer sequences: GAPDH_FWD, 5′-GAA GGT GAA GGT CGG AGT C-3′ and GAPDH_REV, 5′-GAA GAT GGT GAT GGG ATT TC-3′. The GAPDHTM molecular beacon probe was designed to contain the following sequence: 5′-FAM-CAA GCT TCC CGT TCT CAG CCT-BBQ-3′. The reactions were carried out as previously described.

*SIVmac and SHIV-1 infection*. SupT-1 cells stably expressing Myr(+)Ank^GAG^1D4 cells were infected with SIVmac239 or SHIV-Bo159N4-p at MOI 1. The culture supernatants were collected at day 7 (D7), D11, and D18 pi. Thereafter, the levels of p27 were determined by the ELISA assay. The copy number of viral genomic RNA present in the cell culture supernatants was evaluated by qPCR at D18

**SUPPLEMENTARY MATERIAL** **Figure S1.** Analysis of Myr(+)Ank^GAG^1D4EGFP expression in SIV- or SHIV-infected SupT1 cells. **Figure S2.** Sequence alignment of the N-terminal domain of the HIV-1 and SIVmac239 CA proteins (NTD^CA^). **Table S1.** The levels of HIV-1 integration in SupT1 cells harvested at day 24 post-infection.

## Supplementary Material

Supplementary Figure S1 Analysis of Myr(+)Ank^GAG^1D4EGFP expression in SIV- or SHIV-infected SupT1 cells.

Supplementary Figure S2 Sequence alignment of the N-terminal domain of the HIV-1 and SIVmac239 CA proteins (NTD^CA^).

Supplementary Table S1 The levels of HIV-1 integration in SupT1 cells harvested at day 24 post-infection.
