# Supplementary figures and images for: Combined Antiviral Therapy Using Designed Molecular Scaffolds Targeting Two Distinct Viral Functions, HIV-1 Genome Integration and Capsid Assembly
Source: Mol Ther Nucleic Acids. 2015 Aug 25;4(8):e249–. doi: 10.1038/mtna.2015.22 (PMC4560793; doi:10.1038/mtna.2015.22)

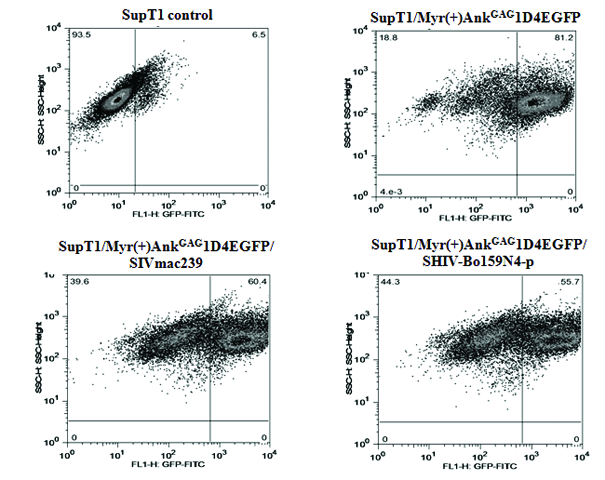

Supplement: Supplementary Figure S1 — Analysis of Myr(+)AnkGAG1D4EGFP expression in SIV- or SHIV-infected SupT1 cells. [file mtna201522x1.tiff]

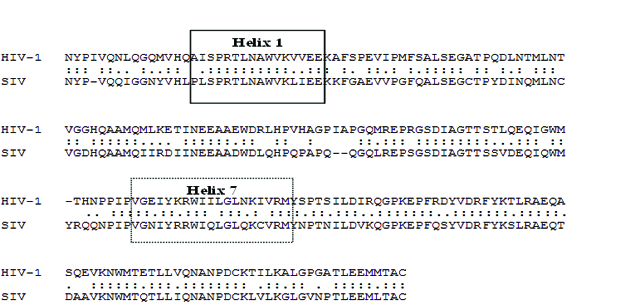

Supplement: Supplementary Figure S2 — Sequence alignment of the N-terminal domain of the HIV-1 and SIVmac239 CA proteins (NTDCA). [file mtna201522x2.tiff]
