# Supplementary material for: Combined Antiviral Therapy Using Designed Molecular Scaffolds Targeting Two Distinct Viral Functions, HIV-1 Genome Integration and Capsid Assembly
Source: Mol Ther Nucleic Acids. 2015 Aug 25;4(8):e249–. doi: 10.1038/mtna.2015.22 (PMC4560793; doi:10.1038/mtna.2015.22)
Supplement: Supplementary Table S1 — The levels of HIV-1 integration in SupT1 cells harvested at day 24 post-infection. [file mtna201522x3.doc]

**Tables**

**Supplementary Table S1. The levels of HIV-1 integration in SupT1 cells harvested at day 24 post-infection ^(a)^**

|  | SupT1 control | | Myr(+)Ank^GAG^1D4 | 2LTRZFP | 2LTRZFP/Myr(+)Ank^GAG^1D4 |
| --- | --- | --- | --- | --- | --- |
| *Alu-gag* qRT-PCR | | 35.8 ± 0.2 | 34.5 ± 0.3 | N/A | N/A |
| *GAPDH* | | 34.6 ± 0.4 | 27.5 ± 0.3 | 26.5 ± 0.6 | 28.5 ± 0.7 |

^(a)^ The levels of HIV-1 integration in SupT1 cells harvested at day 24 post-infection were determined by quantitative real-time PCR (qPCR) performed on host cell DNA extracts, using primers specific to *Alu*-*gag* junctions. The cellular *GAPDH* gene was used as the internal control. The figures given in the table are Ct values, mean ± SD (*n* = 3).
